# Transformed Filaments by Oxygen Plasma Treatment and Improved Resistance State

**DOI:** 10.3390/nano12152716

**Published:** 2022-08-07

**Authors:** Jongmin Park, Jungwhan Choi, Daewon Chung, Sungjun Kim

**Affiliations:** Division of Electronics and Electrical Engineering, Dongguk University, Seoul 04620, Korea

**Keywords:** RRAM, oxygen plasma treatment, XPS, conductive filaments, low power consumption

## Abstract

The simple structure and operation method of resistive random-access memory (RRAM) has attracted attention as next-generation memory. However, as it is greatly influenced by the movement of oxygen atoms during switching, it is essential to minimize the damage and adjust the defects. Here, we fabricated an ITO/SnO_X_/TaN device and investigated the performance improvement with the treatment of O_2_ plasma. Firstly, the change in the forming curve was noticeable, and the defect adjustment was carried out effectively. By comparing the I–V curves, it was confirmed that the resistance increased and the current was successfully suppressed, making it suitable for use as a low-power consumption device. Retention of more than 10^4^ s at room temperature was measured, and an endurance of 200 cycles was performed. The filaments’ configuration was revealed through the depth profile of X-ray photoelectron spectroscopy (XPS) and modeled to be visually observed. The work with plasma treatment provides a variety of applications to the neuromorphic system that require a low-current level.

## 1. Introduction

To meet the demands of consumers for high-density memory, integration technology is constantly evolving. Resistive random-access memory (RRAM) on various metal-oxide materials has drawn attention in recent years because of its high density, fast switching speed, long and stable endurance, efficient power consumption, and excellent compatibility with the CMOS fabrication process [1,2,3]. Resistive switching (RS) is determined by the formation and rupture of conductive filaments and is generally divided into electrochemical metallization memory (ECM) affected by the ionization process of active metal [4,5,6] and valence change memory (VCM) affected by the redox reaction of metal-oxide materials [7,8,9,10]. Therefore, the ability to suppress and regulate the growth of conductive filaments may be one of the ways to promote the development of RRAM devices.

Among metal-oxide materials, tin oxide (SnO_X_) has a wide bandgap, and thus serves as a switching layer in storage-class memory. Particularly, one of the common forms of SnO_X_, tin monoxide (SnO), shows a fundamental bandgap of 3.4 eV and maintains a metastable condition in ambient temperature that makes it easy to form a conductive filament consisting of Sn vacancies and O interstitials [11]. Those ions drift according to the direction of the electric field and determine the states of the device. The RS behavior of as-deposited SnO_X_ formed through sputter has already been proven in the literature [12,13,14,15,16], but better RS characteristics can be achieved using DC-sputtering without the assistance of the annealing process.

As mentioned above, as the conductive filaments are generally composed of defects, such as Sn vacancies or O interstitials, the defect adjustment has a great impact on the switching behavior. There are representative methods for controlling the defect such as annealing treatment, which changes the phase of crystalline [17,18,19,20], and plasma treatment using chemical gases, like Ar [21,22,23,24,25], O_2_ [26,27,28], and N_2_ [29,30]. Far from an annealing process that can be engaged with the thermal budget issue with the CMOS fabrication process, plasma treatment that causes only small damage on the surface is certainly a useful processing technique. We used O_2_ gases for generating the plasma and succeeded in achieving effective results by applying the treatment before depositing the upper electrode.

Here, we used two kinds of ITO/SnO_X_/TaN devices, one with plasma treatment and one without treatment, to measure the electrical properties. We observed the difference in the forming process and fundamental I–V curves affected by the plasma treatment. Further, memory properties, such as retention and endurance, were confirmed. Owing to the O_2_ plasma treatment, the resistance value of the device was increased, thus the current flow was suppressed. We also confirmed that the device performance was not degraded through about 200 cycles of the DC endurance and about 10^4^ s of the data retention. The influence of the O_2_ plasma treatment was ascribed through the X-ray photoelectron spectroscopy (XPS) depth profile, and we visually modeled the shape of filaments on the basis of analysis. The influence of the O_2_ plasma treatment was ascribed through the X-ray photoelectron spectroscopy (XPS) depth profile, and we visually modeled the shape of filaments on the basis of analysis [31].

## 2. Experiments

The Si substrate with the thermally grown SiO_2_ of 300 nm was prepared after the pre-sputtering of TaN (GMEK Korea Inc.). Before depositing thin films, we conducted the cleaning with the chemical products of acetone, isopropyl alcohol, and deionized water. Cleaning was performed for 10 min after immersing the substrate in each chemical product, the ultrasonic cleaner was used, and the blowing work was conducted through an N_2_ gun. The Sn metal target (99.99%, 3 inches) was used to deposit SnO_X_ with DC power. Ar gas of 10 sccm and O_2_ gas of 5 sccm were injected after the main chamber reached the base pressure of 1 μTorr. To prevent contamination, we conducted the pre-sputtering about 10 min before the deposition. The thickness of 50–60 nm was well deposited, and after plasma treatment was performed on the surface of the SnO_X_. The oxygen gas of 100 sccm, pressure of 50 mTorr, power of 50 W, and time of 5 min were set as the treatment conditions. For comparison, the device excluding the treatment was also fabricated. After the patterning, the ITO (90:10 wt %, 99.99%) was deposited in the following condition: DC power of 90 W, 10 sccm of Ar gas, 1 sccm of O_2_ gas, and the others were set the same as the previous deposition. Lastly, the lift-off was performed to finish the fabrication. The film information, such as the thickness and atomic bonding, was investigated through field emission scanning electron microscope (FE-SEM, Hitachi S-4800) and X-ray photoelectron spectroscopy (XPS, Nexsa). The depth profile of the XPS showed thin film information located below the surface. Various electrical properties were confirmed using the semiconductor parameter analyzer (Keithley 4200-SCS).

## 3. Results and Discussion

**Figure 1:**
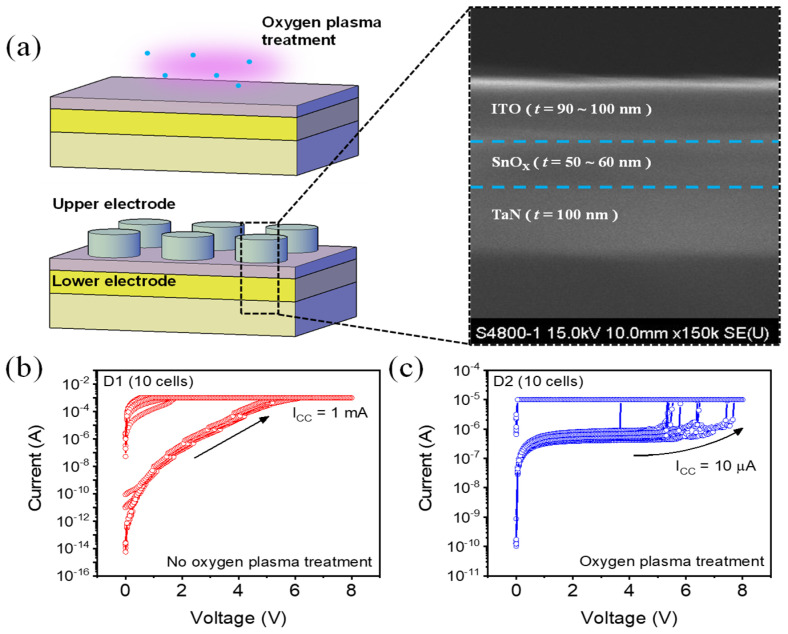
(**a**) The schematic image and the scanning electron microscope (SEM) image of the ITO/SnO_X_/TaN device. (**b**) The forming process of the ITO/SnO_X_/TaN (D1) device started at a low current and formed at 1 mA. (**c**) Forming process of ITO/SnO_X_-oxygen plasma treatment/TaN (D2) device, which started at a higher current and performed at 10 μA. They are measured from 10 different cells.

The formation of filaments has a great role in the switching principle, and the components of filaments are the basis for dividing RRAM into electrochemical metallization mechanism and valence change mechanism (VCM). As the active metals (e.g., Ag or Cu) are not used, the mechanism of the ITO/SnO_X_/TaN device is based on the VCM. As a valid method for controlling the shape of filaments, plasma treatment is widely studied and we are confident that plasma treatment using oxygen gas can have a great effect on VCM switching. Figure 1a shows the schematic image of the oxygen plasma treatment created after SnO_X_ deposition and the pattern formation created thereafter. The thickness information for each layer is shown by the SEM image located next to the schematic image. Figure 1b,c show the respective forming process of ITO/SnO_X_/TaN (D1) and ITO/SnO_X_-after oxygen plasma treatment/TaN (D2). As the amount of defect in the pristine state is more present in D2, the current at a low electric field flowing in the forming process is higher in D2. Even if the current was higher in the forming process, the compliance current (I_CC_) required by D2 from the filaments is 10 μA, which is 100 times lower than 1 mA in D1. We confirmed that oxygen plasma treatment not only affects the amount of defect, but also affects the shape of filaments.

**Figure 2:**
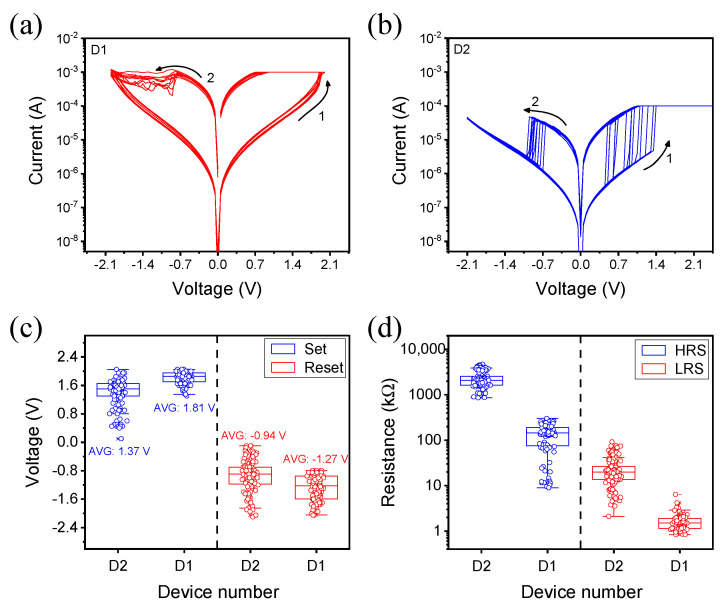
Current–voltage curves of (**a**) the D1 device and (**b**) the D2 device. (**c**) The operating voltage of the set process and reset process are decreased in common. (**d**) A resistance state formed on each device; an increasing aspect is observed after conducting the oxygen plasma treatment.

Figure 2a,b show the 20 different switching curves measured in each device. While we set the same value of ICC on the D1 device, the ICC of 100 μA was set on D2 for smooth operating. The current flowing in D1 gradually increases, while the current in D2 rises sharply after reaching the set voltage. Not only did the switching type change considerably, but also the resistance state and operating voltage changed under the influence of the oxygen plasma treatment. The parameters related to switching are statistically analyzed using 140 switching curves measured from seven different cells. The set and reset voltage decrease by 0.3 V on average in common, but the dispersion increases, as shown in Figure 2c. As shown in Figure 2d, the resistance states are increased by about 10 times and show a reduction in power consumption at the same read voltage of 0.1 V. In addition, it affects the on/off ratio for improving the reliability of the device. The minimum value of the on/off ratio calculated from the box charts in Figure 2d is extended from 1.4 to 9.3. This suggests that oxygen plasma treatment can reduce power consumption and increase switching reliability.

**Figure 3:**
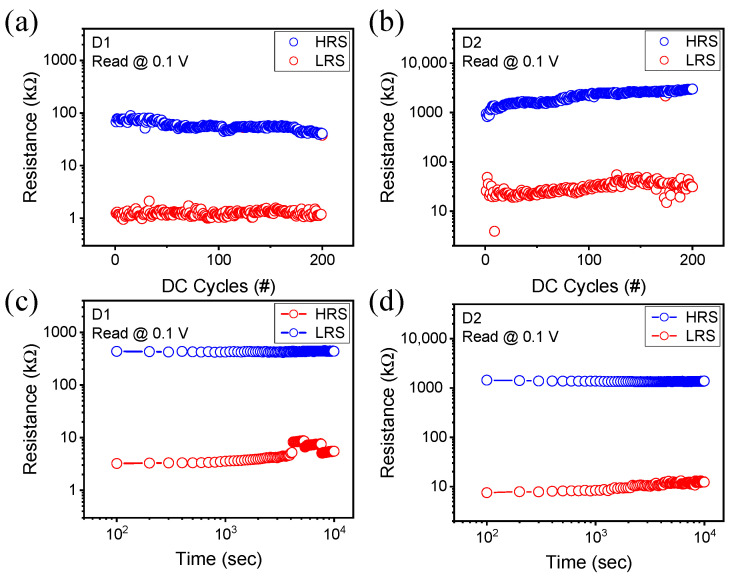
Endurance in (**a**) the D1 device and (**b**) the D2 device measured through the repetitive DC voltage application. Retention in (**c**) the D1 device and (**d**) the D2 device measured at room temperature for 10^4^ s.

To check the performance of RRAM changed by oxygen plasma treatment, the DC voltage is repeatedly swept to confirm the endurance. As shown in Figure 3a, the low-resistance state (LRS) degrades according to the application of repetitive voltage and the on/off ratio of D1 decreases from 60 to 30. However, D2 maintains a constant on/off ratio of 70 or more during the 200 voltage cycles in Figure 3b. Although different ICCs were applied, a voltage range of the same magnitude from −2.1 V to 2.5 V and a read voltage of 0.1 V were used. Retention is measured for 10^4^ s using a voltage of 0.1 V at room temperature, and it is confirmed that resistance states are maintained stably. In both D1 and D2, the high-resistance state (HRS) is maintained more stably than that of the LRS owing to the LRS degradation over time. However, the on/off ratio maintains more than 100 times even after 10^4^ s, which ensures sufficient reliability.

**Figure 4:**
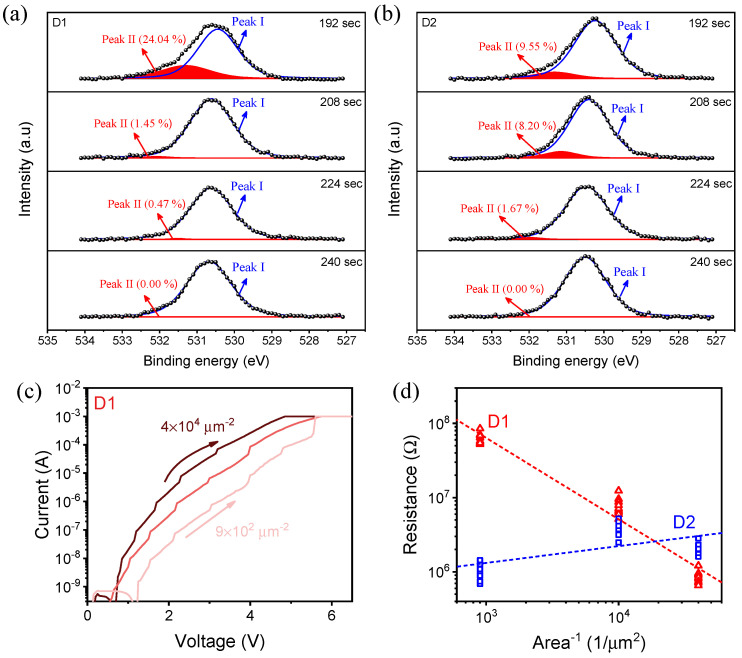
O 1s spectra of (**a**) the D1 device and (**b**) the D2 device. 192/208/224/240 s indicate the region of SnO_X_ and each peak corresponds to the metal-oxide bonding and the oxygen vacancies. (**c**) Forming curves of the D1 device measured in different electrode areas. (**d**) Area-dependent property under the effect of oxygen plasma treatment; 10 different cells in each area were measured.

The reason for the increase in resistance, decrease in operating voltage, and change in the forming process is investigated through the X-ray photoelectron spectroscopy (XPS) depth profile of each device. The depth profile in Figure 4 is conducted by penetrating from the upper electrode and lower electrode. The etch time of 192/208/224/240 s indicates the region of SnO_X_. O 1s core-level spectra are analyzed using Shirley background and the line shape of GL (30). Peak I and peak II corresponded to the metal-oxide bonding and V_O_, respectively. Peak II shows lower intensity than peak I, meaning that the ratio of V_O_ is lower than the oxygen lattice. Chen, P.H. et al. reported that ITO has a superior oxygen storage capacity that induces a high ratio of V_O_ [32]. The device in this paper also shows high peak II intensity in the upper electrode, meaning that a higher ratio of V_O_ is agglomerated in the ITO interface. As shown in Figure 4a,b, it can be confirmed that the internal V_O_ was adjusted through oxygen plasma treatment. Although the V_O_ at the ITO interface, which is directly affected by oxygen plasma, decreased from 24.04% to 9.55%, the proportion of V_O_ is about five times higher with an etch time of 208 s. The oxygen plasma treatment serves to penetrate the V_O_ deeply into the SnO_X_. V_O_ adjusted as follows also leads to a difference in the area-dependent property. Figure 4c shows forming curves of the D1 device in different electrode areas. It shows the proportional relationship between current flow and area size. Besides, Figure 4d with 10 different cells measured in each electrode area shows that the area-dependent property was not strongly confirmed in the D2 device compared with the D1 device. This suggests that the filaments induced throughout the entire electrode were locally remodeled.

**Figure 5:**
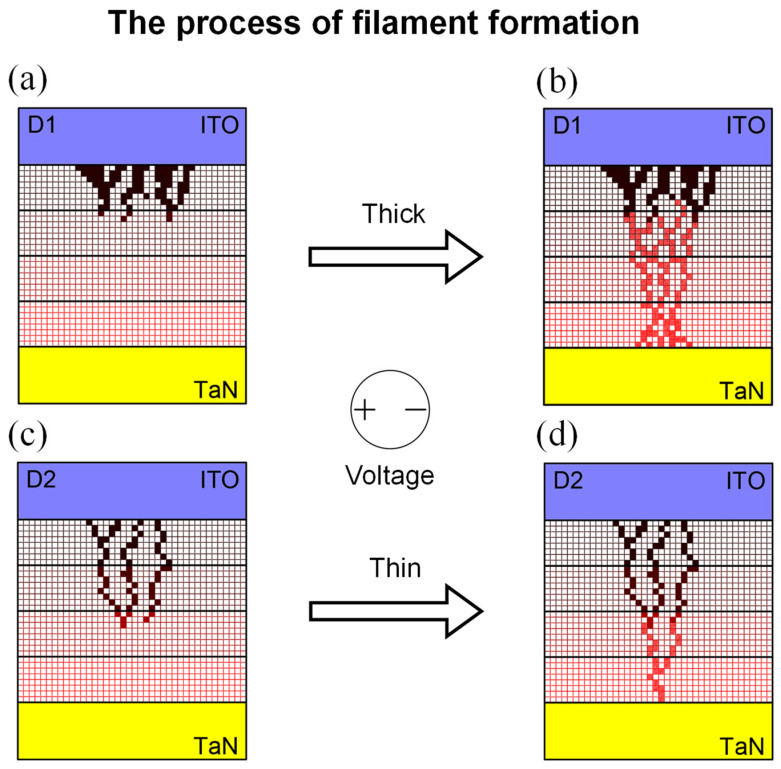
The formation process filaments are modeled on the XPS depth data. (**a**) Pristine state of D1. (**b**) After providing the forming voltage on D1. (**c**) Pristine state of D2. (**d**) After providing the forming voltage on D2. Dark red dots are oxygen vacancies located before electrical stimulation, while bright red dots are oxygen vacancies formed after providing forming voltage.

The appearance of the filaments in Figure 5 is modeled based on the above XPS data, and it is agglomerated at the ITO interface. As shown in Figure 5a,c, corresponding to the pristine states of respective D1 and D2, lumped filaments are formed in D1. while thin and long filaments are formed in D2. Even if it is thin and long, the overall amount of defect is higher in D2 because the current level in the forming process reflects the amount of defect in the pristine state. Filaments penetrating from the upper electrode to the lower electrode are formed after providing the forming voltage in Figure 5b,d. We confirm that the shape of filaments is not collapsed as different sizes of ICC can be applied. As shown in Figure 2c above, the thin filaments formed maintaining the existing shape increase the dispersion of the operating voltage. On the other hand, a flowing current in D2 decreases because the resistance is proportional to the length and inversely proportional to the cross-sectional area. Besides, XPS analysis of indium was conducted to discuss the filament configuration. Proceeding the same as the O 1s core level by listing according to the etch time, there was no clear impact with the plasma treatment. Thus, we concluded that the composition of the filaments was mainly due to the non-lattice oxygen.

## 4. Conclusions

Here, we fabricated the two devices of ITO/SnO_X_/TaN to compare the effect of the oxygen plasma treatment. The effectiveness of the treatment was verified through the comparison of I–V curves in memory function and XPS analysis. The forming curve was dramatically transformed, and the resistance was increased enough to qualify it as a low-power consumption device. It showed that the memory properties of retention and endurance were improved without any degradation. We further considered the deformation of the filaments utilizing the analysis of the O 1s core-level spectra each time. In conclusion, as the visually modeled filament shows, we determined that the plasma treatment using oxygen gas is a very suitable method to control the current level of RRAM. The application on the resistive switching mechanism and XPS spectra in this work could provide a meaningful inspiration to device fabrication.

## Data Availability

Not applicable.
